# Pinpointing Cancer Sub-Type Specific Metabolic Tasks Facilitates Identification of Anti-cancer Targets

**DOI:** 10.3389/fmed.2022.872024

**Published:** 2022-03-23

**Authors:** Shuaishi Gao, Ziwei Dai, Hanyu Xu, Luhua Lai

**Affiliations:** ^1^Center for Quantitative Biology, Academy for Advanced Interdisciplinary Studies, Peking University, Beijing, China; ^2^Department of Biology, School of Life Sciences, Southern University of Science and Technology, Shenzhen, China; ^3^BNLMS, College of Chemistry and Molecular Engineering, Peking University, Beijing, China; ^4^Peking-Tsinghua Center for Life Sciences, Peking University, Beijing, China; ^5^Research Unit of Drug Design Method, Chinese Academy of Medical Sciences, Beijing, China

**Keywords:** cancer metabolism, metabolic network, metabolic task, multi-objective, synthetic lethality

## Abstract

Metabolic reprogramming is one of the hallmarks of tumorigenesis. Understanding the metabolic changes in cancer cells may provide attractive therapeutic targets and new strategies for cancer therapy. The metabolic states are not the same in different cancer types or subtypes, even within the same sample of solid tumors. In order to understand the heterogeneity of cancer cells, we used the Pareto tasks inference method to analyze the metabolic tasks of different cancers, including breast cancer, lung cancer, digestive organ cancer, digestive tract cancer, and reproductive cancer. We found that cancer subtypes haves different propensities toward metabolic tasks, and the biological significance of these metabolic tasks also varies greatly. Normal cells treat metabolic tasks uniformly, while different cancer cells focus on different pathways. We then integrated the metabolic tasks into the multi-objective genome-scale metabolic network model, which shows higher accuracy in the *in silico* prediction of cell states after gene knockout than the conventional biomass maximization model. The predicted potential single drug targets could potentially turn into biomarkers or drug design targets. We further implemented the multi-objective genome-scale metabolic network model to predict synthetic lethal target pairs of the Basal and Luminal B subtypes of breast cancer. By analyzing the predicted synthetic lethal targets, we found that mitochondrial enzymes are potential targets for drug combinations. Our study quantitatively analyzes the metabolic tasks of cancer and establishes cancer type-specific metabolic models, which opens a new window for the development of specific anti-cancer drugs and provides promising treatment plans for specific cancer subtypes.

## Introduction

Metabolic reprogramming is one of the hallmarks of tumorigenesis (1) Understanding the metabolic changes in cancer cells provides attractive therapeutic targets, and new strategies for cancer therapy (2). Altered metabolic activity is vital for sustaining uncontrolled proliferation, evasion of growth suppression, resistance of cell death and the metastasis to other areas. Cancer cells have many metabolic characteristics. In order to provide enough energy source for rapid proliferation, cancer cells involve a shift from mitochondrial metabolism toward glycolysis, even in the presence of oxygen, which is called Warburg effect (3).

The metabolic states are not the same in different cancer types or subtypes, even within the same sample of solid tumors. Metabolic reprogramming is largely based on genetic changes and environment (4, 5), and those different reprogramming routes contribute to metabolic heterogeneity. Many researches, including transcriptomics (6), proteomics (7) and metabolomics (8), show metabolic heterogeneity across cancer types. Although in most cancers, nucleotide synthesis and glycolysis are upregulated, others, like oxidative phosphorylation, vary a lot (6). Cancer subtypes also display diverse metabolic phenotypes. For instance, triple negative breast cancers (TNBCs) rely more on glucose and glutamine uptake than ER+ breast cancers (9). Understanding metabolic heterogeneity is of significance for identifying metabolic vulnerabilities susceptible to therapeutic targeting (2).

Taken into consideration of all those metabolic objectives, it is impossible to simply synthesize one key general criterion for all cancer types, even subtypes that originate from the same tissue. In comparison of experimental and model-predicted flux, Schuetz et al. demonstrated that the combination of two or three metabolic objectives and modeling by Flux Balance Analysis (FBA) offers a better performance for the explanation of flux distribution in microbe modeling (10, 11). The theory of evolutionary trade-offs can shed light on the biological significance of multi-task optimization, as cells need to perform multiple tasks that all contribute to fitness in gene expression space (12, 13). Using a multi-objective genome-scale metabolic model is a reasonable approach to improve the accuracy and prediction ability of genome-scale metabolic network modeling in evolutionary terms (14).

In order to understand the heterogeneity of cancer cells, we construct a multi-objective metabolic model (MOMM) using transcriptomics data of different cancer types. Specifically, we studied breast cancer, lung cancer, digestive organ cancer, digestive tract cancer, and reproductive cancer. To evaluate our methodology, we applied MOMM in the prediction of cell viability. We implemented MOMM to predict synthetic lethal target pairs, and many of the top-ranking pairs have been reported as lethal target pairs in previous experimental studies. Our analysis showed that the number of activated synthetic lethal target pairs is a good marker of patient survival time. This study opens a new window for the development of anti-cancer drugs and provides promising treatment plans for cancer.

## Materials and Methods

### Pre-processing of Data

The transcriptomics data used in this study comes from TCGA (15). Cancer types include breast cancer, lung cancer, digestive organ cancer, digestive tract cancer, and reproductive organ cancer.

(1) Breast cancer contains 1,215 samples, with four PAM50 subtypes and normal types, as well as unknown types. Among them, the basal subtype of breast cancer contains 142 samples, the luminal A subtype contains 423 samples, the luminal B subtype contains 194 samples, the HER2 subtype contains 67 samples, and the normal cell contains 137 samples.
(2) Lung cancer contains 1,129 samples, with two pathological types and normal types. Among them, lung adenocarcinoma contains 517 samples, lung squamous cell carcinoma contains 502 samples, and normal type contains 110 samples.
(3) Digestive organ cancer contains 651 samples, and there are three cancer subtypes and normal types. Among them, liver cancer contains 373 samples, bile duct cancer contains 36 samples, pancreatic cancer contains 179 samples, and normal cell contain 63 samples.
(4) digestive tract cancer contains 444 samples, and there are two cancer subtypes and normal types. Among them, colon cancer contains 288 samples, rectal cancer contains 95 samples, and normal type has 51 samples.
(5) Reproductive organ cancer contains 874 samples, and there are four cancer subtypes and normal types. Among them, ovarian cancer contained 308 samples, endometrial cancer contained 177 samples, cervical cancer contained 305 samples, uterine carcinosarcoma contained 57 samples, and normal cells contained 27 samples.

The genome-wide metabolic network used in the model is the Recon 1 model supplemented by Duarte et al. (16). On top of this, we imposed additional constraints on the intake of 20 amino acids and important metabolites (Supplementary Table 4). The network used in the simulation contains 4,924 metabolic reactions (reversible reactions are disassembled and counted as 2 reactions, so that the flow rate is non-negative, which is convenient for subsequent calculations) and 2,767 metabolites. By comparing the existence of metabolic genes in the metabolic network in the transcriptome data, we obtained a total of 1,442 metabolic-related gene expression profile data for subsequent analysis.

### Inference of Metabolic Tasks Using Pareto-Tasks Inference Method

We used Pareto-tasks inference method to interpret the number of metabolic tasks (17). By using the default algorithm, we used three criteria to evaluate the propriate number of tasks. (1) Simplicity: The greater the number of metabolic tasks, the greater the explained variance and the more information it contains, but more complex the model will be. We used the Elbow plot to find the best trade-off task number between information content and model complexity. (2) Interpretability, we used the t-ratio test, the smaller the *P*-value. The more explanatory the model is for biological significance. (3) Stability: The average error value of the vertex position of the multi-objective model is calculated by the bootstrapping method. The smaller the average error value, the stronger the model stability.

### Evaluation of Multi-Objective Metabolic Model

The relationship between gene expression and survival probability is represented by hazard ration (HR). Genes with HR > 1 means that their low expression corresponds to high survival probability; genes with HR < 1 means that their high expression corresponds to high survival probability. We select genes whose objective function value are within [*Obj*_*min*_ + 0.1*(*Obj*_*wt*_−*Obj*_*min*_), *Obj*_*wt*_] after their knockout to calculate their receiver operating characteristic (ROC) curve, where *Obj*_*wt*_ is the wild-type objective function value before gene knockout simulation, *Obj*_*min*_ is the minimum value of the objective function in all gene simulations, that is, the most affected state.

### Integration of Metabolic Tasks and Genome-Scale Metabolic Network Model

The metabolic tasks that predicted by Pareto task inference (ParTI) method are represented by the metabolic gene expression level. Integrating the metabolic tasks into the genome-scales metabolic network is essentially integrating the gene expression to the flux model.

First, we mapped the gene expression level to metabolic enzyme expression level. In the genome-scale metabolic network, the relationship between reactions and genes and proteins is connected by gene-protein-reaction (GPR) relationships, which are Boolean expressions between transcripts, proteins, and the corresponding reactions. Isozymes are represented by OR relationship, and we used the sum of the expression of isozymes as the total expression of the enzyme. If the protein contains multiple subunits, it is represented by AND relationship. We used the minimum value of the subunits to represent the total expression of the enzyme.


$$r_{1}\;g_{1}\,\text{OR}\,g_{2}\;\mathrm{sum}\,\left(g_{1},g_{2}\right)$$

$$r_{2}\;g_{3}\,\text{AND}\,g_{4}\;\min\,\left(g_{3},g_{4}\right)$$

Then, we integrated the enzyme expression to flux by E-Fmin method (18),


(1) $$\text{max}\,\sum_{i}w_{i}\,v_{i}$$

Subject to,


(2) S=⁢0

(3) $$v_{L}\leq \nu \leq \nu_{U}$$

(4) $$\varepsilon \leq v_{b\,i\,o\,m\,a\,s\,s}$$

Where *w*_*i*_ is the function of enzyme expression, $w_{i}=\bar{r_{i}}-1$, $\bar{r_{i}}$ is the normalized enzyme expression. *v*_*i*_ is the flux of the i*^th^* reaction. S is the stoichiometric matrix (m× n), m represents the number of metabolites, n represents the number of reactions. ν is the sum of *n* flux, *v*_*L*_ and ν_*U*_ are the lower and upper bound of ν. *v*_*biomass*_ is the flux of biomass, its lower bound ε depends on the real situation. Through the prediction of the model, we can calculate the optimal flux distribution of the corresponding metabolic task.

### *In silico* Simulation of Gene Knockouts

We used minimization of metabolic adjustment (MOMA) to calculate the flux distribution after knockout (19). Essentially, we use this method to find new flux distribution which is the closest to the original flux distribution in new feasible area.


(5) $$\text{D}\,\left(\text{w},\text{x}\right)=\sqrt{\sum_{i=1}^{N}{\left(w_{i}-v_{i}\right)}^{2}}$$

Where *w* is the original flux distribution, *w*_*i*_ is the ith flux, *v* ∈ ϕ_*j*_ is the flux distribution after knockouts, *v*_*i*_ is the ith flux after gene modification, N is the number of fluxes. D(*w*,*v*) represents the Euclidean distance between *w* and *v*. We want to find *v* which let *D* minimizes. So we turn (5) into (6), i.e., minimize,


(6) $$\text{f}\,\left(\text{x}\right)=L\cdot v+\frac{1}{2}\,v^{T}\,Q\,v$$

Where *L* and *Q* are the linear and quadratic parts of the objective function, the length of *L* is N, the size of *Q* is *N* × *N*, *v^T^* is the transpose of *v*. Minimizing (6) is equivalent to minimizing the quadratic formula inside the rood, and the constant term after expansion can also be ignored. Therefore, minimizing *D* is equivalent to minimizing *f(v)*. If only the optimal solution exists, and the feasible region is not empty after adjustment, we can always find a solution to this optimization problem. The uniqueness of the solution is guaranteed by the convexity of *f(v)*.

### Identification of Synthetic Lethality Pairs

We simulated the paired knockouts of all genes in the metabolic network, and used MOMA to calculate the flux distribution afterward. According to “highest single agent” synergy model (20), i.e., synthetic lethality pair represents the combined effect outperforms either its single component. Here, we exclude “transport” and “exchange” related reactions, and only consider intracellular reactions.

We introduce Impact Score (IS) to represent the impact of the cell after knockouts. *IS*=(*obj*_*max*_−*obj*_*val*_)/(*obj*_*max*_−*obj*_*min*_), where *obj*_*max*_ represents the maximum of the objective function, which is the original value without gene modification; *obj*_*min*_ represents the minimal objective function value in all the combined knockout simulations; *obj*_*val*_ represents the corresponding objective function value after the specific gene pair knockouts. We choose gene pairs with *IS*≥ 0.5 in the following analysis.

For survival analysis in synthetic lethality pairs evaluation, we choose the breast cancer GEO data set, with 1,809 patients (21). And we use the subtype classification method derived by Mihály, etc., to category the sample (22).

## Results

### The Dominant Metabolic Tasks for Cancers

We used multi-objective metabolic model (MOMM) to answer the questions of how many and what are the dominant metabolic tasks for a specific type of cancer. We assume that every single cell should be in a Pareto-optimal situation in order to survive. Here, we used TCGA gene expression data by RNA-seq (data was obtained from UCSC Xena)^1^ to build the model. Only metabolism-related expression profile data were used. The metabolism-related genes were defined as the genes that are involved in the metabolic reactions in Recon 1, a commonly used genome-scale metabolic network (16), and at the same time, have expression records in the TCGA data set (Details of data were shown in Supplementary Table 1).

According to the theory of evolutionary trade-offs between tasks, whole-cell gene expression can fall into a low-dimensional polytope whose vertices are the optimal points in each task alone (12, 13). We therefore inquired whether a low-dimensional polytope (e.g., line, triangle, or tetrahedron) could enclose the metabolism-related gene expression data. We used the ParTI method to fit the metabolism-related transcriptomics data into a low-dimensional polyhedron, in which each vertex, i.e., archetype, represents an optimal phenotype for a single metabolic tasks (17). This approach was used in many biological contexts, including population and single-cell level gene expression (13, 23).

The more the metabolic tasks are, the more accurate the model, but less simplicity. To identify the dominant metabolic tasks that can cover as much information as possible without being overly complicated, we set three criteria: simplicity, interpretability, and stability (see section “Materials and Methods”). Simplicity represents the complexity level of metabolic tasks model and is analyzed by explained variability of different number of archetypes. Interpretability represents the biological significance of the calculated metabolic tasks, i.e., the description of the data of these metabolic tasks. Stability requires that the metabolic tasks are not changed along with the sample size changes, and is calculated by bootstrapping.

**FIGURE 1:**
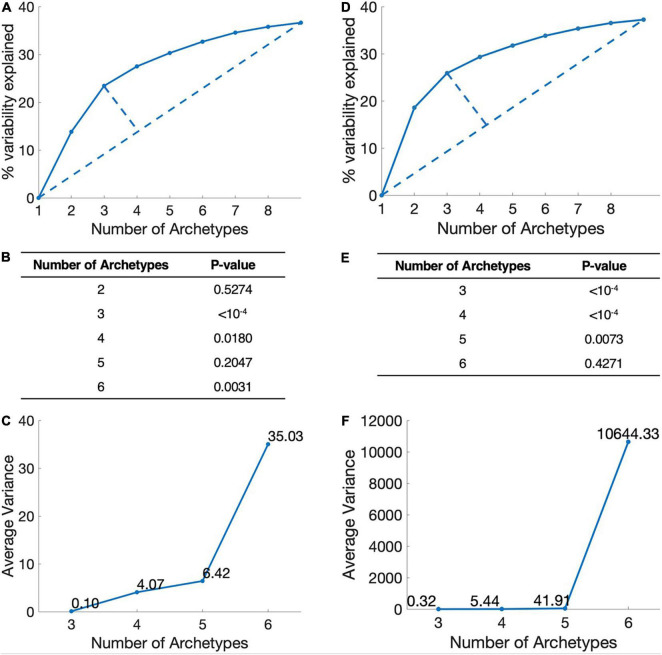
Determination of the number of metabolic tasks. **(A)** Simplicity, the elbow plot of the number of metabolic tasks and the explained variance in breast cancer. The point with the largest distance is the best trade-off point between the amount of information and model complexity. This figure show three-task model is the best. **(B)** Interpretability, *P*-value of t-ratio test for different metabolic target numbers of breast cancer. **(C)** Stability, the average variance of the vertices position of breast cancer. **(D–F)** Lung cancer.

By applying these criteria, we found that the combination of three dominant metabolic tasks is the best representation for breast cancer (Figures 1A–C). Although the combination of four metabolic tasks also works comparably, it adds more model complexity and is less stable.

Applying the same criteria, we found that the combination of three metabolic tasks is the best representation for lung cancer (Figures 1D–F). From the perspective of simplicity and stability, models with three tasks are the optimal modeling direction. However, from an interpretability point of view, models with three or four metabolic tasks are similar. Therefore, we choose three tasks for the follow-up analysis of lung cancer.

We also analyzed the metabolic tasks in digestive organ cancers (include liver cancer, bile duct cancer, and pancreatic cancer), digestive tract cancers (include colon cancer and rectal cancer) and reproductive system cancers (include ovarian cancer, endometrial cancer, cervical cancer, and uterine carcinosarcoma). The models for digestive organ cancers, digestive tract cancers and reproductive system cancers contain four, three, and three dominating metabolic tasks, respectively (Details were given in Supplementary Material).

### The Biological Significance of Metabolic Tasks

Based on the estimation of the number of metabolic tasks, we further analyzed the biological significance of metabolic tasks, including their corresponding subtype information and biological functions. We mainly focus on the breast cancer and lung cancer. In terms of the propensity of different subtypes of metabolic tasks, we calculate the degree of enrichment of the subtypes around the metabolic task. We inferred the significance of the metabolic task through the gene sets that enriched around the metabolic task. Through the analysis of the cancer subtype enrichment, we found that different cancer subtypes have different metabolic task tendencies, and their biological significance is also quite different.

**FIGURE 2:**
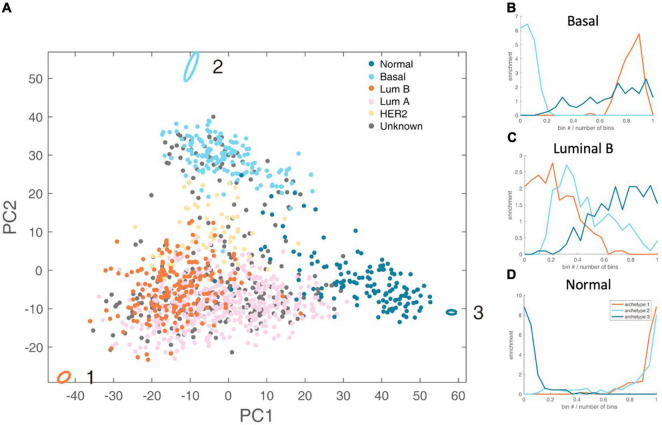
Schematic representation of the three-task of breast cancer and enrichment analysis of breast cancer subtypes. **(A)** The vertices in the figure represent the best metabolic tasks. The ellipse represents the error value calculated by the bootstrap method. The dots represent patient samples, and the color represents the clinical molecular subtype of PAM50. See the legend at the top right. **(B–D)** The enrichment distribution relative to the three metabolic tasks of basal, luminal B, and normal type. The horizontal axis represents the Euclidean distance ranking of the data bin (see section “Materials and Methods” for definition) and the metabolic task, and the vertical axis is the degree of enrichment, which is defined as the ratio of the density of the subtype in this bin to the overall density.

By analyzing the PAM50 subtypes of breast cancer, including luminal A subtype, luminal B subtype, basal subtype, HER2 subtype, and normal subtype, we found that luminal B, basal and normal subtype have clear metabolic task tendency (Figure 2).

Luminal B accounts for about 35% of breast cancer, and is estrogen receptor (ER) positive, and either human epidermal growth factor receptor 2 (HER2) positive or negative (24). We found that luminal B has dominated energy metabolism (Hypergeometric test *P*-value <1×10^–4^). There are only two enriched kyoto encyclopedia of genes and genomes (KEGG) metabolic pathways of Luminal B subtype, namely steroid synthesis and oxidative phosphorylation (Supplementary Table 2). That is, the gene expression levels of these two metabolic pathways are significantly higher than other metabolic tasks. Steroid hormones can exert their mitogenic effects by binding to estrogen, progesterone and androgen receptors (25). Targeting steroid synthesis pathway to control the development of luminal B cancer has been clinically applied, such as the treatment using anastrozole, a competitive inhibitor of androgen synthesis (26). Oxidative phosphorylation is related to energy production and cell metabolism intermediate production. Experiments at the cellular level have shown that luminal B cell line is more active in oxidative phosphorylation, and inhibiting the upstream regulatory pathway of oxidative phosphorylation, i.e., mTOR pathway, can reduce cell respiration, while has no effect on basal subtype (27).

Basal subtype is also known as triple-negative breast cancer, the overlap of the two subtypes is about 70–80% (28). Basal subtype accounts for about 10–20% of breast cancer with poor survival rate (29). Basal subtype tends to accomplish rapid proliferation (Hypergeometric test *P*-value < 1 × 10^–4^). It enriches six KEGG metabolic pathways, including folic acid synthesis, oxidative phosphorylation, glyoxylic acid and dicarboxylic acid metabolism, etc (Supplementary Table 2). Folic acid is a carbon donor for one-carbon metabolism. Folic acid supports the production of NADPH, nucleotide production and methylation. Cancer cells up-regulate the folic acid metabolism pathway that is related to DNA production and cell growth (30). Basal subtype also enriches oxidative phosphorylation pathway. Clinical studies have shown that targeting oxidative phosphorylation provides an effective way to treat basal subtype (31, 32). Glyoxylic acid and dicarboxylic acid pathways are highly expressed in basal breast cancer (33).

Normal cells tend to fulfill the metabolic tasks other than cancer tasks (Hypergeometric test *P*-value < 1 × 10^–4^). Interestingly, there are 60 gene sets enriched for normal cells metabolic tasks, far exceeding luminal B and basal. The metabolic tasks of normal cells should be maintaining metabolic homeostasis, such as the balance of redox potential and the stable rhythm of the circadian clock.

**FIGURE 3:**
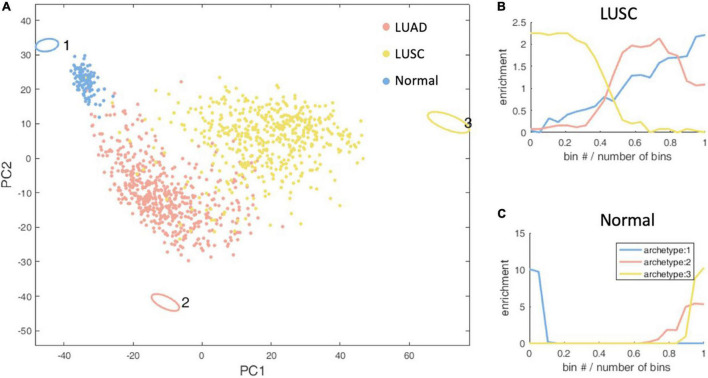
Schematic representation of the three-task of lung cancer and enrichment analysis of lung cancer subtypes. **(A)** The vertices in the figure represent the best metabolic tasks. The ellipse represents the error value calculated by the bootstrap method. The dots represent patient samples, and the color represents the clinical molecular subtype of PAM50. See the legend at the top right. **(B,C)** The enrichment distribution relative to the three metabolic tasks of LUSC and normal type. The horizontal axis represents the Euclidean distance ranking of the data bin (see section “Materials and Methods” for definition) and the metabolic task, and the vertical axis is the degree of enrichment, which is defined as the ratio of the density of the subtype in this bin to the overall density.

We performed the same analysis for lung cancer (Figure 3). There are two pathological types and the normal type of lung cancer samples. The pathological types are lung adenocarcinoma (LUAD) and lung squamous cell carcinoma (LUSC).

Through the enrichment analysis of the KEGG metabolic pathway for lung cancer metabolic tasks, we found that LUSC cancer cells tend to complete the metabolic task 3 (Hypergeometric test *P*-value <1×10^–4^), but with no significantly enriched KEGG metabolic pathways. However, the metabolism task 1, which normal cells tend to accomplish (Hypergeometric test *P*-value < 1 × 10^–4^), has 36 KEGG metabolic pathways significantly enriched. As no enriched KEGG metabolic pathways were found in LUSC, in order to understand the biological significance of the metabolic task of cancer cells, we alternatively conducted enrichment analysis of the REACTOME metabolic pathways and found that the metabolic tasks of LUSC enriched in six pathways, including basic immunoglobulin interactions, vascular wall cell surface interactions, and amino acids synthesis and transamination, glycolysis, Gastrin-CREB signaling pathways through PKC and MAPK, and purine nucleoside monophosphate biosynthesis, which as a whole, are related to biomass production and energy production. Normal cellular metabolism tasks are enriched in 33 REACTOME pathways, and are related to the maintenance of tissue function and homeostasis. No highly enriched pathways are found for LUAD.

The reason that there are more metabolic pathways enriched in normal cells than cancer cells is largely because normal cells need to maintain function and body homeostasis, while cancer cells do not have clear physiological functions, but only to meet the monolithic requirements of proliferation and migration.

### Multi-Objective Metabolic Model Outperforms Classical Objective Function in Gene Knockout Simulations

We use MOMM to simulate gene knockout to predict cell state. In order to verify the predictive power of the model, we compare the MOMM prediction results with the maximization of biomass production model.

We first use Kaplan–Meier online analyzer^2^ to analyze the effect of gene expression on the survival probability of breast cancer patients. This online tool collected the relationship between the expression levels of 22,277 genes in 1,809 patients and the prognosis of cancer patients (34). We selected genes that have effect on cells after knockout, and compared the ROC curves of the two models. The selection criteria of genes and the calculation method of ROC curve are shown in section “Materials and Methods.”

**FIGURE 4:**
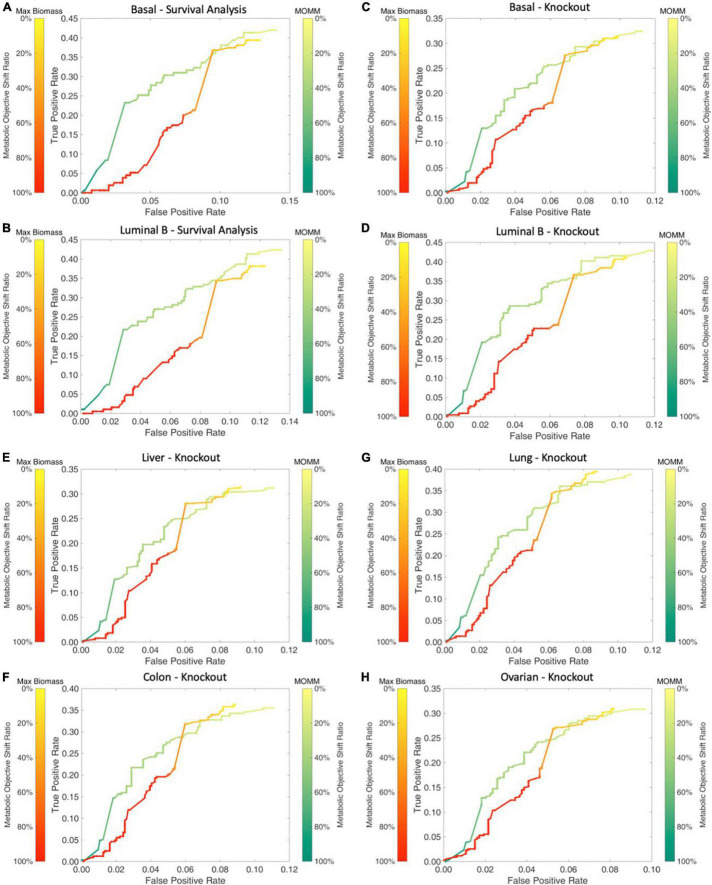
Comparison between MOMM and the maximization of biomass model. **(A,B)** The survival comparison of basal and luminal B, the green line represents MOMM, and the red line represents the biomass model. The shade of the color represents the degree of influence on the objective function, the darker the color, the stronger the influence, and the 10% influence degree of the original function is used as the threshold. **(C–H)** Comparison of the accuracy of MOMM and the biomass model on the CRISPR-Cas9 knockout gene database DepMap.

We used breast cancer basal and luminal B subtype specific MOMM to simulate the impact of all metabolic gene knockout on cells, and the ratio of the deviation degree of the objective function value was used to express the degree of the gene knockout effect. We found that our model performed better than the maximize biomass model (Figures 4A,B).

Subsequently, we also used the CRISPR-Cas9 gene knockout database of cell line data to further compare MOMM and biomass model. We obtained the Boolean relationship matrix between cell lines and gene essentiality from the DepMap database^3^. The essentiality means that after knocking out with CRISPR-Cas9, it has a significant impact on the adaptability of the cell. We define that for the same type of cell line, if a gene is necessary in at least one cell line, this gene is also necessary for this type of cancer. See the Supplementary Table 3 for the cell type classification of cell lines.

We used the model to predict the basal and luminal B subtype, lung adenocarcinoma in lung cancer, liver cancer in digestive organ cancer, colon cancer in digestive tract cancer, and ovarian cancer in reproductive organ cancer. We found that MOMM outperforms the maximization of biomass production in all these cancer types (Figures 4C–H).

### Exploration of Potential Synthetic Lethality Gene Pairs of Breast Cancer Subtypes

Synthetic lethality refers to the phenomenon that when two genes are interfered at the same time, it will cause cell death, while single ones will not be lethal (35). The interference includes loss of function mutation, RNA interference (RNAi), and drug therapy.

We used MOMM to perform pairwise knockout simulations for all reactions, to calculate the value of the objective function after double knockout, and used the highest single agent method to determine whether it is a valid combination (see section “Materials and Methods”). For luminal B subtype, there are 9,854 synthetical lethality pairs (SLPs). For basal subtype, there are 11,198 SLPs. Tables 1, 2 list the top 20 SLPs for basal and luminal B, respectively.

**TABLE 1:** The top 20 SLPs of basal breast cancer.

Reaction A	Reaction B	IS^a^
Glutathione oxidoreductase	Cytochrome c oxidase, mitochondrial Complex IV	1.000
L-Lactate dehydrogenase, cytosolic/mitochondrial	Ubiquinol-6 cytochrome c reductase, Complex III	0.989
Cytochrome c oxidase, mitochondrial Complex IV	Acetyl-CoA carboxylase	0.987
Hydrogen peroxide synthesis (NADPH dependent)	Cytochrome c oxidase, mitochondrial Complex IV	0.980
Phosphofructokinase	Cytochrome c oxidase, mitochondrial Complex IV	0.979
Triose-phosphate isomerase forward	Cytochrome c oxidase, mitochondrial Complex IV	0.975
Glutathione peroxidase, mitochondria forward	Cytochrome c oxidase, mitochondrial Complex IV	0.970
Cytochrome c oxidase, mitochondrial Complex IV	Acetyl-CoA C-acetyltransferase, mitochondrial forward	0.969
Inorganic diphosphatase	Cytochrome c oxidase, mitochondrial Complex IV	0.969
Pyruvate dehydrogenase	Cytochrome c oxidase, mitochondrial Complex IV	0.967
L-lactate dehydrogenase backward	Ubiquinol-6 cytochrome c reductase, Complex III	0.964
Acetyl-CoA C-acetyltransferase backward	Cytochrome c oxidase, mitochondrial Complex IV	0.945
Cytochrome c oxidase, mitochondrial Complex IV	Acetoacetyl-CoA:acetate CoA-transferase forward	0.944
Ribulose 5-phosphate 3-epimerase forward	Cytochrome c oxidase, mitochondrial Complex IV	0.939
ATP synthase (four protons for one ATP)	Acetyl-CoA carboxylase	0.937
Ubiquinol-6 cytochrome c reductase, Complex III	Acetyl-CoA C-acetyltransferase, mitochondrial forward	0.933
Retinol dehydrogenase (all-trans, NADPH) forward	Cytochrome c oxidase, mitochondrial Complex IV	0.931
Fructose-bisphosphate aldolase forward	Cytochrome c oxidase, mitochondrial Complex IV	0.930
Nucleoside-diphosphate kinase (ATP:dTDP) forward	Cytochrome c oxidase, mitochondrial Complex IV	0.925
Fatty-acid–CoA ligase forward	Cytochrome c oxidase, mitochondrial Complex IV	0.923

*^a^IS, Impact Score, definition is given in section “Materials and Methods”.*

**TABLE 2:** The top 20 SLPs of luminal B breast cancer.

Reaction A	Reaction B	IS
Glutathione oxidoreductase	Cytochrome c oxidase, mitochondrial Complex IV	1.000
L-Lactate dehydrogenase, cytosolic/mitochondrial	Ubiquinol-6 cytochrome c reductase, Complex III	0.975
Hydrogen peroxide synthesis (NADPH dependent)	Cytochrome c oxidase, mitochondrial Complex IV	0.968
Triose-phosphate isomerase forward	Cytochrome c oxidase, mitochondrial Complex IV	0.966
Glutathione peroxidase, mitochondria forward	Cytochrome c oxidase, mitochondrial Complex IV	0.962
Cytochrome c oxidase, mitochondrial Complex IV	Acetyl-CoA C-acetyltransferase, mitochondrial forward	0.960
Inorganic diphosphatase	Cytochrome c oxidase, mitochondrial Complex IV	0.958
L-lactate dehydrogenase backward	Ubiquinol-6 cytochrome c reductase, Complex III	0.956
Pyruvate dehydrogenase	Cytochrome c oxidase, mitochondrial Complex IV	0.955
Cytochrome c oxidase, mitochondrial Complex IV	Acetyl-CoA carboxylase	0.951
Acetyl-CoA C-acetyltransferase backward	Cytochrome c oxidase, mitochondrial Complex IV	0.937
Fructose-bisphosphate aldolase forward	Cytochrome c oxidase, mitochondrial Complex IV	0.935
Cytochrome c oxidase, mitochondrial Complex IV	Acetoacetyl-CoA:acetate CoA-transferase forward	0.935
Phosphofructokinase	Cytochrome c oxidase, mitochondrial Complex IV	0.932
ATP synthase (four protons for one ATP)	Acetyl-CoA carboxylase	0.923
Ubiquinol-6 cytochrome c reductase, Complex III	Acetyl-CoA C-acetyltransferase, mitochondrial forward	0.921
Retinol dehydrogenase (all-trans, NADPH) forward	Cytochrome c oxidase, mitochondrial Complex IV	0.920
Nucleoside-diphosphate kinase (ATP:dTDP) forward	Cytochrome c oxidase, mitochondrial Complex IV	0.915
Nucleoside-diphosphate kinase (ATP:dUDP) forward	Cytochrome c oxidase, mitochondrial Complex IV	0.913
Cytochrome c oxidase, mitochondrial Complex IV	Aspartate transaminase forward	0.907

We found that the top 1 and 2 SLPs in both basal and luminal B are the same, one is glutathione oxidoreductase (also named as glutathione reductase, GR) and cytochrome c oxidase, mitochondrial complex IV (COX), the other is L-lactate dehydrogenase (LDH) and ubiquinol-6 cytochrome c reductase, mitochondrial complex III (UQCR).

Glutathione reductase is an antioxidant that catalyzes the reduction of glutathione disulfide to glutathione, and its high expression is related to the resistance of cancer cells to oxidative stress (36). COX is the last enzyme in the mitochondrial respiratory chain. It is the oxygen receptor of the respiratory chain and catalyzes the reduction of oxygen to water. Anthracyclines such as daunomycin (DAU) and doxorubicin (DOX) are breast cancer chemotherapy drugs, and their side effects are that they can cause cardiomyopathy during long-term treatment (37, 38). Both DAU and DOX can inhibit COX and promote the production of ROS, and the inhibitory effect is related to the dose of the drug (39). Flavonoids are inhibitors of glutathione reductase (40). Studies have shown that flavonoid inhibitors can reduce the cardiotoxic side effects of anthracyclines (41). Breast cancer cell line experiments have shown that the combined use of flavonoid drug quercetin and anthracycline DOX has better anti-tumor effect on highly aggressive breast cancer cells than the effect of single use, and weakens the side effects of DOX on non-tumor cells (42). Our results provide another theoretical basis for the combined use of flavonoids and anthracyclines in cancer treatment.

Lactate dehydrogenase catalyzes the conversion of lactic acid to pyruvate, and is usually highly expressed in breast cancer (27). LDH is located in the mitochondrial matrix. UQCR is overexpressed in breast cancer, and its knockdown can reduce the aggressiveness of breast cancer (43). Jeong et al. found that after inhibiting LDH in Chinese hamster ovary (CHO) cells, the respiration rate of the cells increased and they were more sensitive to UQCR inhibitor Antimycin A. After inhibiting both LDH and UQCR, cell activity decreased more than UQCR alone was inhibited (44).

We also counted the frequency of metabolic reactions in the SLPs, and found that mitochondrial energy metabolism-related enzymes occur most frequently, including ATP synthase (four protons for one ATP), acetyl-CoA carboxylase, cytochrome c oxidase, NADH dehydrogenase, ubiquinol-6 cytochrome c reductase, pyruvate dehydrogenase, acetate CoA-transferase. This suggests the importance of mitochondrial-related enzymes in combination medication. In fact, the inhibition of mitochondria already has some applications in tumor treatment. Wang et al. combined treatments targeting mitochondria and radiotherapy to reduce the growth of multidrug resistant tumors without significant systemic toxicity (45). Inhibition of mitochondria can also overcome dose toxicity. In addition to reduce cardiotoxicity of anthracyclines, it can also reduce the systemic toxicity of glycolysis inhibitors. Cheng et al. used mitochondrial inhibitors and glycolysis inhibitor 2-deoxy-D-glucose treatment of basal subtype of human breast cancer xenograft model, and found that the tumor size was significantly reduced, and the kidney, liver, and heart had no obvious morphological changes (46).

Other lethal combinations for which there is no direct experimental or clinical evidence may provide new guidelines for combination drug design. Most metabolic pathways also play important roles in normal cells, so the design of combination drugs may reduce the dosage of single drugs and avoid serious side effects.

We also compared the ranking of SLPs in single-target models. In most cases, the two targets in one top-ranking SLP pair do not rank high at the same time in single-target analysis. For example, combination of GR and COX of the Basal subtype is the top 1 SLP, while GR ranks 214th and COX ranks 4th in Basal single-target analysis. For the Luminal B subtype, combination of GR and COX are also the top 1 SLP, while GR ranks 139th and COX ranks 5th in the single-target analysis of Luminal B.

### The Number of Activated Synthetic Lethality Gene Pairs as a Marker for Relapse-Free Survival

We analyzed the correlation between SLPs and breast cancer patient survival to evaluate the model. Patients data come from Györffy et al. (21). It should be pointed out that we did not select TCGA data that used to infer metabolic tasks to avoid circular reasoning. The data set contains 230 basal patients and 265 luminal B patients. We define activated SLPs as SLPs with low expression of gene A and high expression of gene B. We calculated the number of activated SLPs in patients with different subtypes. When the number of activated SLPs in the patient is higher than three-quarters of the number of activated SLPs in all patients of this subtype, we define that the patient has a high level of activated SLPs. When the patient’s activated SLPs is less than a quarter of all patients of this subtype, we define that the patient has a low level of activated SLPs.

**FIGURE 5:**
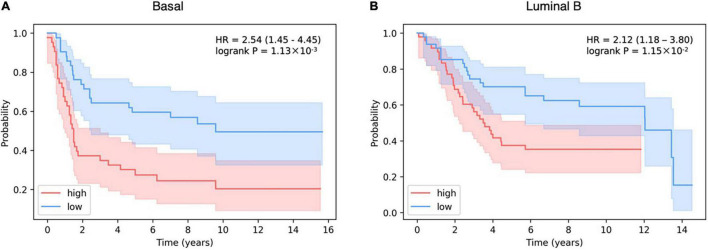
The survival curve of the high and low SLP level. **(A)** Basal subtype; **(B)** Luminal B subtype.

We found that the number of activated SLPs can be used as an indicator of relapse free survival (RFS). Patients with higher survival probability have less activated SLPs, possibly due to less severe disorders of the patients’ metabolic network. While patients with lower survival probability have more activated SLPs (Basal: HR = 2.54, *P*-value = 1.13 × 10^–3^; Luminal B: HR = 2.12, *P*-value = 1.15 × 10^–2^; Figure 5).

**FIGURE 6:**
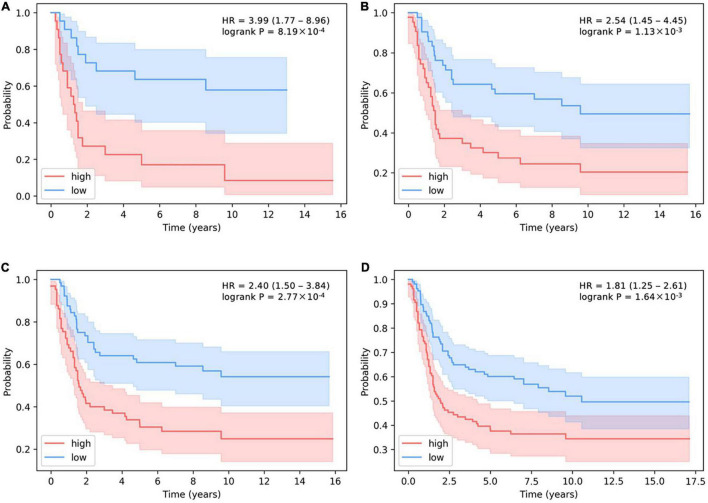
The determination of the relationship between the threshold of activated SLP levels and survival prediction in basal subtype. **(A–D)** The thresholds for the number of high-level SLPs are the top 10, 20, 30, and 50%, and the thresholds for the number of low-level SLPs are the bottom 10, 20, 30, and 50% of all patients.

In addition, the stricter the threshold for judging the number of activated SLPs, the more obvious the predictive effect on survival (Figure 6). HR > 1 means that patients are more likely to have cancer recurrence. The larger the HR is, the more dangerous situation the patients are in. When we decrease the threshold for distinguishing the number of activated SLPs, the value of HR will be lower, which proves the predictive effect of our model. If we choose 10% as threshold, i.e., high-level SLPs are the top 10% and low-level SLPs are the bottom 10% of all patients, HR is 3.99 (*P*-value = 8.19 × 10^–4^). HR is 2.53 (*P*-value = 1.13 × 10^–3^) when 20% is the threshold; HR is 2.40 (*P*-value = 2.77 × 10^–4^) when 30% is the threshold; and HR is 1.81 (*P*-value = 1.64 × 10^–3^) when 50% is the threshold.

## Discussion

Due to the lack of fluxomics data, we used transcriptomics data instead to defer the metabolic tasks. Although transcriptomics data and fluxomics data are positively correlated, there are discrepancies due to the presence of post-transcriptional regulation and translation process regulation (47, 48). More experimental fluxomics data in the future will help to improve the current analysis.

Metabolic heterogeneity not only exists among different cancer subtypes, but also among patients within the same cancer subtype. When patient-specific transcriptomics data are available, personalized drug targets and treatment plans can be predicted. Along with the rapid development of single cell technologies, heterogeneity of cancer cells within one cancer tissue of a patient can be analyzed and treatment plans considering the diversity and spatial arrangement of the cancer cells can be given. Moreover, the tumor-associated immune cells also have metabolic reprogramming during tumor progression to facilitate the escape of tumor cells from immune surveillance (49, 50). Our metabolic tasks analysis method may also be used to uncover the metabolic regulators in the tumor-associated immune cells in future studies.

Metabolic reprogramming is a key feature and vulnerable point of cancer cells. Predictive mathematical modeling can incorporate multiple levels of data to enable a better understanding of metabolic reprogramming in cancer cells and uncover therapeutic targets (51–53).

The heterogeneity of cancer cell metabolism due to different metabolic tasks increases the complexity of research and cancer drug discovery. In this study, we used ParTI method to analyze the metabolic tasks of breast cancer, liver cancer, digestive organ cancer, digestive tract cancer and reproductive system cancer. We found that different cancer types have different metabolic task tendencies with different biological implications. The metabolic tasks of cancer cells are more “uniform” compared to normal cells. The metabolic tasks of different sub-types of the same cancer are also different. For example, the luminal B subtype of breast cancer is more prone to energy metabolism, and the basal subtype is more prone to rapid proliferation. The anticancer target prediction by MOMM outperforms the conventional biomass maximization objective as evaluated by patient survival analysis.

## Data Availability Statement

Publicly available datasets were analyzed in this study. This data can be found here: http://cancergenome.nih.gov/.

## Author Contributions

SG and LL conceived and designed the project, developed the model, analyzed the data, and wrote the manuscript. ZD and HX contributed analytic tools. LL supervised the whole project. All authors contributed to the article and approved the submitted version.

## Conflict of Interest

The authors declare that the research was conducted in the absence of any commercial or financial relationships that could be construed as a potential conflict of interest.

## Publisher’s Note

All claims expressed in this article are solely those of the authors and do not necessarily represent those of their affiliated organizations, or those of the publisher, the editors and the reviewers. Any product that may be evaluated in this article, or claim that may be made by its manufacturer, is not guaranteed or endorsed by the publisher.

## Footnotes

1
http://xena.ucsc.edu/

2
http://kmplot.com/

3
https://score.depmap.sanger.ac.uk/
