# Supplementary material for: Pinpointing Cancer Sub-Type Specific Metabolic Tasks Facilitates Identification of Anti-cancer Targets
Source: Front Med (Lausanne). 2022 Mar 23;9:872024. doi: 10.3389/fmed.2022.872024 (PMC8984102; doi:10.3389/fmed.2022.872024)
Supplement: Supplementary file 1 [file Data_Sheet_1.PDF]

frontiers

Supplementary Material

1 Supplementary Results

1.1 The dominant metabolic tasks for other cancer types

For digestive organ cancer, including liver cancer, bile duct cancer and pancreatic cancer. It is difficult to draw a deterministic conclusion from the above analysis, because from the perspective of simplicity, the two goals perform better, but from the interpretability point of view, the three and four metabolic tasks are better, and the five metabolic tasks are the best in terms of stability. And the four tasks are also relatively stable (Supplementary Figure 1A-C). Since the cancer types contained in digestive organ cancer originate from different tissues, and there may be other subtypes in different cancer types, the metabolic target is more complicated. In subsequent analysis, the number of metabolic tasks can be judged in combination with other cell properties, for example, by analyzing the tendency of cancer types to metabolic targets. We found that only in the three-target model, cancer types have a significant metabolic task propensity (Hypergeometric test P-value <1×10-4), liver cancer cells and normal cells tend to complete different metabolic targets, so the three-objective model can be used to analyze the digestive organ cancer.

For digestive tract cancer, including colon cancer, rectal cancer. For simplicity, four-task model is the best, but for interpretability and stability, three-task model is the best (Supplementary Figure 1D-F). In terms of discrimination between metabolic targets and different cancer types, only in the four-task model, colon cancer has a significant metabolic target tendency (Hypergeometric test P-value$<1\times10^{-4}$).

For reproductive system cancer, including ovarian cancer, endometrioid cancer, cervical cancer and uterine carcinosarcoma. The four-task model is better from of deterministic criteria, but three-task model is relatively good in terms of interpretability and stability (Supplementary Figure 1G-I). However, only in the four-task model, ovarian cancer has a significant metabolic target tendency (Hypergeometric test P-value$<1\times10^{-4}$).

However, for digestive organ cancer, digestive tract cancer and reproductive system cancer that cover more complex cancer types than breast cancer and lung cancer, more data are necessary. In this study, we add other characteristic when choose that best number of metabolic tasks that is at least one cancer type (*e.g.* colon cancer in digestive tract cancer) that shows significant propensity towards a metabolic task.

1.2 Analysis of cell metabolic state represented by classical metabolic objective function

Essentially, MOMM is a bottom-up method that we first know the state of the cell, then infer the corresponding metabolic task. The classic FBA method is top-down, that is, assuming cells in a certain state, then constructing the model according to the hypothetical objective equation. We try to compare the biological similarities between the top-down classical FBA method and MOMM. We listed 12 possible objective equations according to literature (Supplementary Table 5). We divided the target equation into four categories, namely cell building blocks, energy, redox, and efficiency.

Next, we compared the Spearman correlation coefficient of the flux distribution obtained by MOMM and the classical construction method (Supplementary Table 6). It is worth noting that here we only compare goals relate to maximization, because the optimal flux distribution relate to minimization must be zero. In addition, in order to avoid the influence of extremely small flux, we set all fluxes that less than $10^{-6}(\mathrm{mmol/(gDW \cdot h)})$ to 0. We found that maximization of biomass production has the highest correlation towards both luminal B and basal subtype. While maximization of mitochondrial ATP production has the highest correlation towards normal cell, followed by maximization of NADPH production.

As the actual metabolic target of the cell cannot be directly expressed by the classical objectives, we further combined the classical metabolic objectives into three objectives combination. We use the ε-constraint method to find the Pareto solution of the objectives' combination. In simple terms, this method transforms the three-objective optimization problem into a set of single-objective linear programming problem, and then optimizes it and obtains Pareto optimal solution under these three objectives (e.g. the maximization of NADPH production under the constraints of biomass production and ATP production). We compared the Pareto optimal metabolic flux distribution generate from classical objectives' combination with the metabolic flux distribution of breast cancer. It is worth noting that at least one metabolic task in the three combination is to maximize, otherwise the flux distribution of the optimal solution will all be zero. In the selection of combination, we make at least one metabolic target the classic metabolic target with the highest correlation in single task comparison. For example, for luminal B task, the combination must include the maximization of biomass production. We found that the correlation between the metabolic flux distribution obtained by MOMM and the combination of the classic metabolic target is related to the weight of the classic metabolic tasks. There are some objectives' combinations are more relevant than the original classic objective. For example, for the basal subtype, maximizing biomass production has the highest correlation. When maximizing biomass production, maximizing mitochondrial ATP production, and minimizing the total intracellular flux, the correlation with the flux distribution predicted by MOMM is improved, from the original 0.580 to the highest 0.914 (Supplementary Figure 2A). When the combination of maximizing biomass production and maximizing ATP hydrolysis and minimizing the total amount of enzymes, the correlation and minimizing the total amount of enzymes, the correlation is not significantly improved, and the highest increase is 0.556 (Supplementary Figure 2B).

1.3 Compare lethal combinations with synthetic lethality database

We analyzed the predicted SLPs with SynLethDB, a comprehensive synthetic lethality database with 19,952 synthetic lethality gene pairs, which integrates Syn-lethality, BioGRID, GenomeRNAi, DE-CIPHER and DAISY. Within SynLenthDB, there are 203 metabolism-related synthetic lethality gene pairs, which means both genes are in the metabolic network. Then we compare the predicted SLPs with these 203 metabolism-related synthetic lethality gene pairs.

We first converted the predicted SLPs into gene pairs through GPR, then compare with SynLethDB. We found that there are 15 gene pairs of Basal subtype in SynLethDB (Fisher's exact test P-value = 1.21×10^{-16}) (Supplementary Table 7), and 12 gene pairs of Lum B in SynLethDB (Fisher's exact test P-value = 4.32×10^{-13}) (Supplementary Table 8).

1.4 Analysis of metabolic pathway interactions based on SLPs

We also explored the relationship between metabolic pathways. In both Basal and Lum B subtype, the genes in oxidative phosphorylation pathway appear the most in the predicted synthetic lethality pairs, and oxidative phosphorylation pathway is more related to nucleotides pathway. Other significant pathways include fatty acid metabolism and glycolysis/gluconeogenesis. Those pathways are important energy or building blocks generated pathways.

2 Supplementary Reference

1. Shlomi T, Benyamini T, Gottlieb E, Sharan R, Ruppin E. Genome-scale metabolic modeling elucidates the role of proliferative adaptation in causing the Warburg effect. *PLoS Comput Biol* (2011) **7**:e1002018. doi:10.1371/journal.pcbi.1002018

2. Budinich M, Bourdon J, Larhlimi A, Eveillard D. A multi-objective constraint-based approach for modeling genome-scale microbial ecosystems. *PLoS One* (2017) **12**:e0171744. doi:10.1371/journal.pone.0171744

3. Mori M, Hwa T, Martin OC, De Martino A, Marinari E. Constrained allocation flux balance analysis. *PLOS Comput Biol* (2016) **12**:e1004913. doi:10.1371/journal.pcbi.1004913

4. Flamholz A, Noor E, Bar-Even A, Liebermeister W, Milo R. Glycolytic strategy as a tradeoff between energy yield and protein cost. *Proc Natl Acad Sci* (2013) **110**:10039–10044. doi:10.1073/pnas.1215283110

5. Jia D, Lu M, Jung KH, Park JH, Yu L, Onuchic JN, Kaipparettu BA, Levine H. Elucidating cancer metabolic plasticity by coupling gene regulation with metabolic pathways. *Proc Natl Acad Sci* (2019) **116**:3909–3918. doi:10.1073/pnas.1816391116

6. Zielinski DC, Jamshidi N, Corbett AJ, Bordbar A, Thomas A, Palsson BO. Systems biology analysis of drivers underlying hallmarks of cancer cell metabolism. *Sci Rep* (2017) **7**:41241. doi:10.1038/srep41241

7. Rafalski VA, Mancini E, Brunet A. Energy metabolism and energy-sensing pathways in mammalian embryonic and adult stem cell fate. *J Cell Sci* (2012) **125**:5597–5608. doi:10.1242/jcs.114827

8. Hackett SR, Zanotelli VRT, Xu W, Goya J, Park JO, Perlman DH, Gibney PA, Botstein D, Storey JD, Rabinowitz JD. Systems-level analysis of mechanisms regulating yeast metabolic flux. *Science* (2016) **354**:aaf2786. doi:10.1126/science.aaf2786

9. Schuetz R, Kuepfer L, Sauer U. Systematic evaluation of objective functions for predicting intracellular fluxes in Escherichia coli. *Mol Syst Biol* (2007) **3**:119. doi:10.1038/msb4100162

3 Supplementary Tables

Supplementary Table 1. Description of transcriptomics data

Type	Subtype	Number of Samples	Number of Metabolism-related Genes
Breast cancer	Basal	142	1,442
	Luminal B	194	
	Luminal A	423	
	HER2	67	
	Unknown	252	
	Normal	137	
Lung cancer	Lung adenocarcinoma	517	
	Lung squamous cell carcinoma	502	
	Normal	110	
Digestive organ cancer	Liver cancer	373	
	Bile duct cancer	36	
	Pancreatic cancer	179	
	Normal	63	
Digestive tract cancer	Colon cancer	288	
	Rectal cancer	95	
	Normal	51	
Reproductive system cancer	Ovarian cancer	308	
	Endometrioid cancer	177	
	Cervical cancer	305	

Uterine carcinosarcoma	57
Normal	27

Supplementary Table 2. KEGG pathways enriched by metabolic tasks

Subtype	Number of enriched KEGG pathways	KEGG pathway
Luminal B	2	Steroid Biosynthesis
		Oxidative Phosphorylation
Basal	6	Glycosphingolipid Biosynthesis Lacto And Neolacto Series
		Glyoxylate And Dicarboxylate Metabolism
		Galactose Metabolism
		Folate Biosynthesis
		Oxidative Phosphorylation
		Epithelial Cell Signaling In Helicobacter Pylori Infection
Normal	60	Tyrosine Metabolism
		Retinol Metabolism
		Phenylalanine Metabolism
		Adipocytokine Signaling Pathway
		Drug Metabolism Cytochrome P450
		Metabolism Of Xenobiotics By Cytochrome P450
		Fatty Acid Metabolism
		Insulin Signaling Pathway

	Proximal Tubule Bicarbonate Reclamation
	Histidine Metabolism
	Dilated Cardiomyopathy
	Ppar Signaling Pathway
	Vascular Smooth Muscle Contraction
	Progesterone Mediated Oocyte Maturation
	Gap Junction
	Tryptophan Metabolism
	Glycerolipid Metabolism
	Melanogenesis
	Starch And Sucrose Metabolism
	Glycolysis Gluconeogenesis
	Oocyte Meiosis
	Steroid Hormone Biosynthesis
	Linoleic Acid Metabolism
	Beta Alanine Metabolism
	Gnrh Signaling Pathway
	Nitrogen Metabolism
	Propanoate Metabolism

	Primary Bile Acid Biosynthesis
	Ascorbate And Aldarate Metabolism
	Calcium Signaling Pathway
	Pyruvate Metabolism
	Glycine Serine And Threonine Metabolism
	Chemokine Signaling Pathway
	Arachidonic Acid Metabolism
	Nicotinate And Nicotinamide Metabolism
	Long Term Depression
	Small Cell Lung Cancer
	Glycerophospholipid Metabolism
	Valine Leucine And Isoleucine Degradation
	Fc Gamma R Mediated Phagocytosis
	Type Ii Diabetes Mellitus
	Citrate Cycle Tca Cycle
	Aldosterone Regulated Sodium Reabsorption
	Vegf Signaling Pathway
	Lysine Degradation
	Butanoate Metabolism

	Fc Epsilon Ri Signaling Pathway
	Pathways In Cancer
	Alanine Aspartate And Glutamate Metabolism
	Glioma
	Peroxisome
	Purine Metabolism
	Alpha Linolenic Acid Metabolism
	Glycosphingolipid Biosynthesis Ganglio Series
	Drug Metabolism Other Enzymes
	Glyoxylate And Dicarboxylate Metabolism
	Porphyrin And Chlorophyll Metabolism
	One Carbon Pool By Folate
	Arginine And Proline Metabolism
	Pentose Phosphate Pathway

Supplementary Table 3. Cell line types in DepMap

**Subtype**	**Cell line**
Basal	CAL51, HCC1806, MDAMB157, HS578T, HCC1395, HCC1143, BT549, MDAMB468, MDAMB436, MDAMB231, HCC1937, DU4475, SUM159PT
Luminal B	CAMA1, MDAMB415, EFM19, HCC1428, HCC1419
Lung Squamous Cell Carcinoma	RERFLCAI, LUDLU1, HCC95, NCIH2170, CALU1, EPLC272H, SKMES1, SW1573, HARA, KNS62, HCC15
Liver Cancer	SNU398, SNU886, HLF, SNU449, LI7, HUH1, JHH4, HUH7, SNU182, SNU761, JHH1, HUH6, JHH5, JHH7, PLCPRF5
Colon Cancer	C2BBE1, COLO201, COLO678, NCIH747, SW837, HCC56, SW1463, NCIH716, HT29, SW620, SNU503, HT55, RKO, LOVO, LS180, SW48, KM12, HT115, DLD1
Ovarian Cancer	ONCODG1, OAW28, COV434, JHOS2, JHOM1, COV318, COV362, SNU840, OV90, EFO21, JHOC5, SNU8, 59M, KURAMOCHI, OVISE, HEYA8, JHOS4, COV644, OVMANA, A2780, OVTOKO, OV7, OVCAR8, RMUGS, CAOV3, MCAS, SKOV3, TOV21G, ES2, EFO27, OVK18, COV504, OVCAR5, COV413A

Supplementary Table 4. Metabolites influx constraints

Metabolite	Lower bound of influx	Upper bound of influx
L-alanine	0	0.05
Glycine	0	0.05
L-Isoleucine	0	0.05
L-Leucine	0	0.05
L-Proline	0	0.05
L-Valine	0	0.05
L-Phenylalanine	0	0.05
L-Tryptophan	0	0.05
L-Tyrosine	0	0.05
L-Aspartic acid	0	0.05
L-Glutamic acid	0	0.05
L-Arginine	0	0.05
L-Histidine	0	0.05
L-Lysine	0	0.05
L-Serine	0	0.05
L-Threonine	0	0.05
L-Cysteine	0	0.05
L-Methionine	0	0.05
L-Asparagine	0	0.05
L-Glutamine	0	0.05
Biotin	0	0.005
Choline	0	0.005
Pantothenate	0	0.005
Folic acid	0	0.005
Nicotinamide	0	0.005
Pyridoxal	0	0.005
Riboflavin	0	0.005
Ammonium sulfate	0	0.005
Inositol	0	0.005
Reduced glutathione	0	0.05

*In reference to RPMI-1640 medium components

Supplementary Table 5. Metabolites influx constraints

Category	Metabolic task	Direction	Reference
Building Blocks	Biomass	Max	(1)
	Intake carbon number	Min	(2)
Energy	ATP hydrolysis	Max	(3)
	Glycolytic ATP	Max	(4)
	Mitochondrial ATP	Max	(5)
Redox	NADH	Min Max	(6)
	NADPH	Min Max	(6)
	ROS	Min	(7)
	5832 DLAT	1737 0.173	0.501
ALDH18A1			
Efficiency	Overall enzyme concentration	Min	(8)
	Overall intracellular flux	Min	(9)

Supplementary Table 6. The Spearman's correlation coefficient between conventional metabolic tasks and MoMM metabolic tasks

	**Archetype 1**	**Archetype 2**	**Archetype 3**
Maximization of biomass production	**0.580**	**0.585**	0.046
Maximization of total ATP hydrolysis	0.057	0.057	0.217

Maximization of total ATP production in glycolysis	0.202	0.201	0.228
Maximization ATP production in mitochondria	0.072	0.072	**0.577**
Maximization of NADH production	0.149	0.147	0.072
Maximization of NADPH production	0.051	0.050	**0.447**

Supplementary Table 7. The synthetic gene pairs in Basal subtype which are also in SynLethDB

GeneA Symbol	GeneA ID	GeneB Symbol	GeneB ID	Disease Score[a]	IS
COX6C	1345	UQCRB	7381	0.200	0.892
ACO2	50	UQCR10	29796	0.200	0.798
FH	2271	UQCRC2	7385	0.164	0.795
CA5A	763	CYP3A4	1576	0.524	0.647
CA5A	763	CYP2C9	1559	0.524	0.646
FLAD1	80308	NDUFS2	4720	0.200	0.642
CA5A	763	CYP3A5	1577	0.524	0.639
NDUFB5	4711	UGP2	7360	0.200	0.632
CA5A	763	CYP1A2	1544	0.524	0.631
CA5A	763	CYP2C19	1557	0.524	0.631
DLAT	1737	SPTLC1	10558	0.164	0.629
CA5A	763	PTGS1	5742	0.524	0.625
DLD	1738	SUCLA2	8803	0.155	0.530
ALDH18A1	5832	DLAT	1737	0.173	0.507
DLAT	1737	PTGS1	5742	0.506	0.506

[a]Disease Score is evaluation score for synthetic lethality pairs in SynLethDB, the higher the score, the higher the credibility

Supplementary Table 8. The synthetic gene pairs in Lum B subtype which are also in SynLethDB

GeneA Symbol	GeneA ID	GeneB Symbol	GeneB ID	Disease Score	IS
CA5A	763	CYP2C9	1559	0.524	0.669
CA5A	763	CYP2C19	1557	0.524	0.660
CA5A	763	CYP2D6	1565	0.524	0.653
CA5A	763	CYP3A5	1577	0.524	0.652
CA5A	763	CYP3A4	1576	0.524	0.638
CA5A	763	PTGS1	5742	0.524	0.638
FLAD1	80308	NDUFS2	4720	0.200	0.636
NDUFB5	4711	UGP2	7360	0.200	0.628
CA5A	763	CYP1A2	1544	0.524	0.622
DLAT	1737	SPTLC1	10558	0.164	0.620
DLD	1738	SUCLA2	8803	0.155	0.511

4 Supplementary Figures

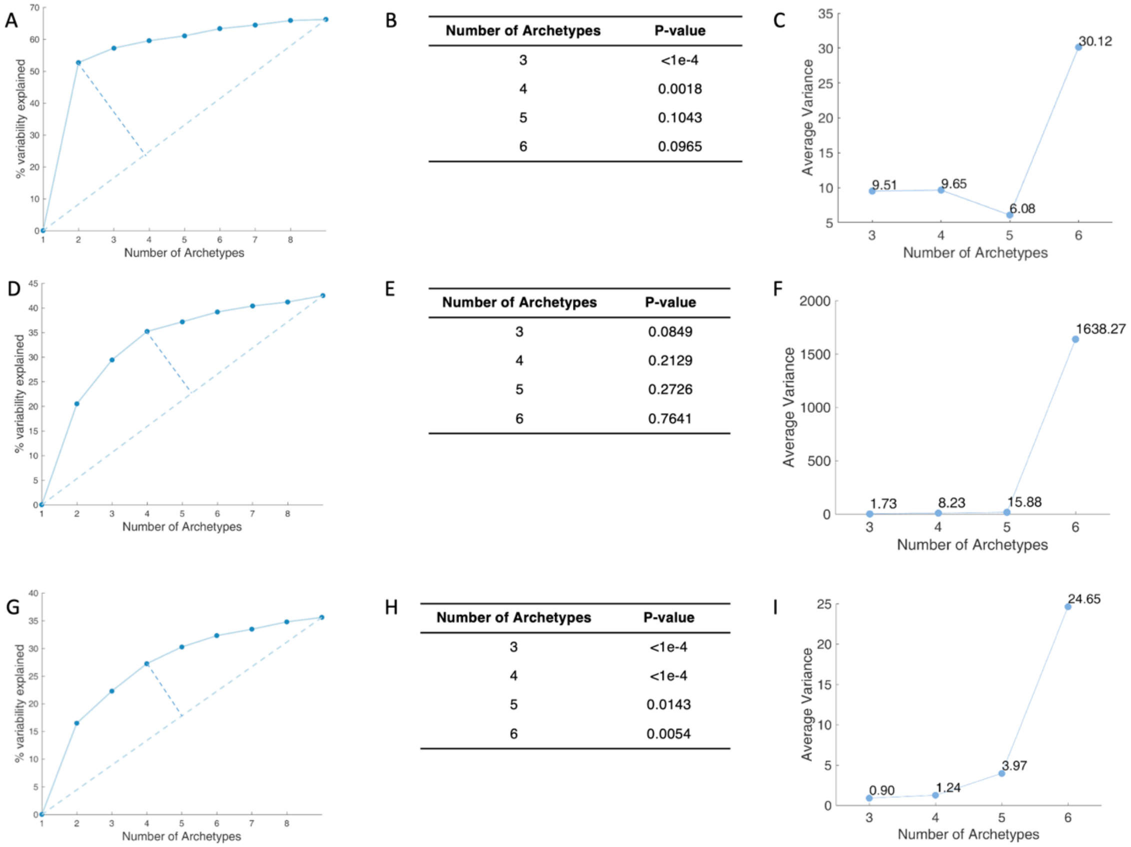

B

Number of Archetypes	P-value
3	<1e-4
4	0.0018
5	0.1043
6	0.0965

E

Number of Archetypes	P-value
3	0.0849
4	0.2129
5	0.2726
6	0.7641

H

Number of Archetypes	P-value
3	<1e-4
4	<1e-4
5	0.0143
6	0.0054

Supplementary Figure 1. Determination of the number of metabolic tasks. **(A)-(C)** Digestive organ cancer. **(A)** Simplicity, the elbow plot of the number of metabolic tasks and the explained variance in breast cancer. The point with the largest distance is the best trade-off point between the amount of information and model complexity. This figure show three-task model is the best. **(B)** Interpretability, P-value of t-ratio test for different metabolic target numbers of breast cancer. **(C)** Stability, the average variance of the vertices position of breast cancer. **(D)-(F)** Digestive tract cancer. **(G)-(I)** Reproductive system cancer.

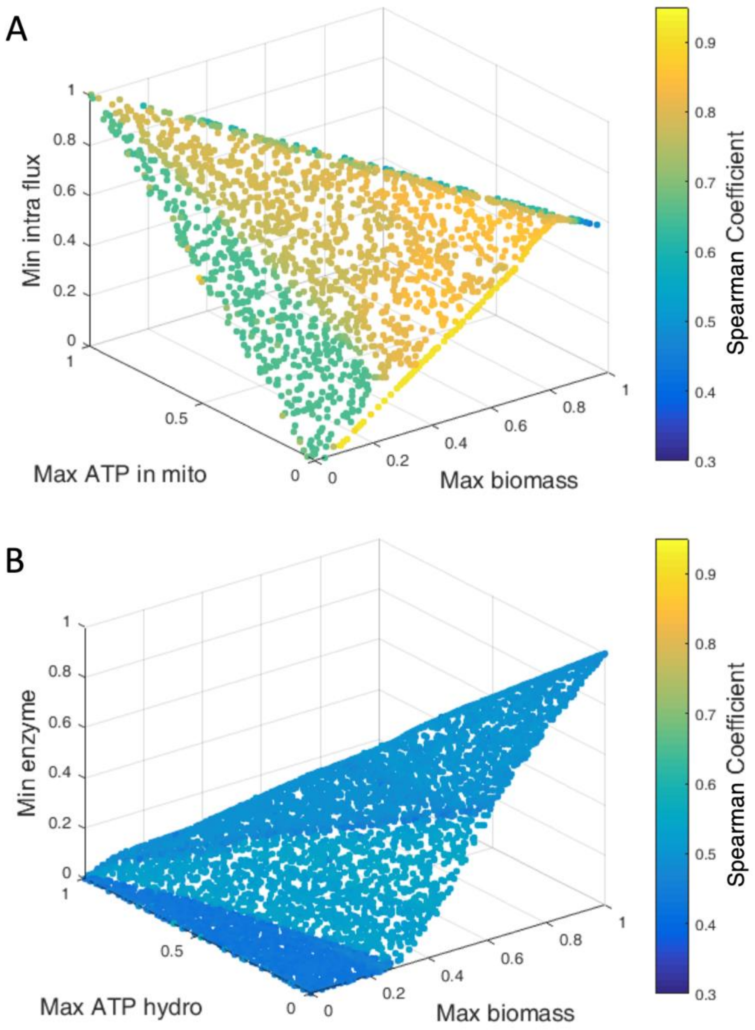

Supplementary Figure 2. Correlation analysis of MoMM breast cancer flux distribution and conventional metabolic tasks, taking the Basal subtype as an example, **(A)** the maximization of biomass production, the maximization of ATP production in mitochondria and the minimization of intracellular flux; **(B)** the maximization of biomass production, the maximization of ATP hydrolysis and the minimization of overall enzyme concentration.

A

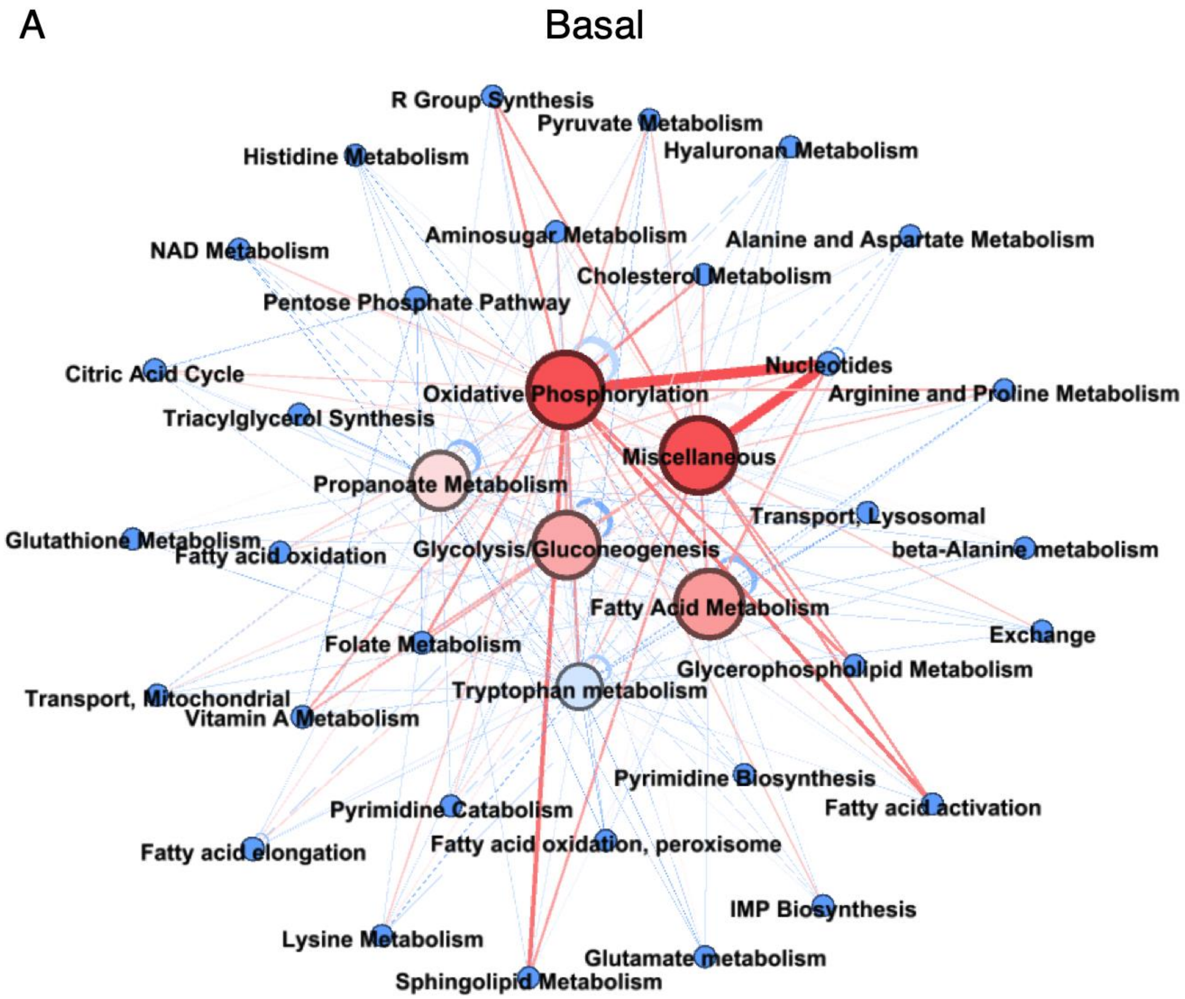
Basal
R Group Synthesis
Pyruvate Metabolism
Hyaluronan Metabolism
Histidine Metabolism
Aminosugar Metabolism
Alanine and Aspartate Metabolism
NAD Metabolism
Cholesterol Metabolism
Pentose Phosphate Pathway
Citric Acid Cycle
Nucleotides
Oxidative Phosphorylation
Arginine and Proline Metabolism
Triacylglycerol Synthesis
Miscellaneous
Propanoate Metabolism
Transport, Lysosomal
Glutathione Metabolism
Fatty acid oxidation
Glycolysis/Gluconeogenesis
beta-Alanine metabolism
Fatty Acid Metabolism
Exchange
Folate Metabolism
Glycerophospholipid Metabolism
Tryptophan metabolism
Transport, Mitochondrial
Vitamin A Metabolism
Pyrimidine Biosynthesis
Pyrimidine Catabolism
Fatty acid activation
Fatty acid oxidation, peroxisome
Fatty acid elongation
IMP Biosynthesis
Lysine Metabolism
Glutamate metabolism
Sphingolipid Metabolism

B Luminal B

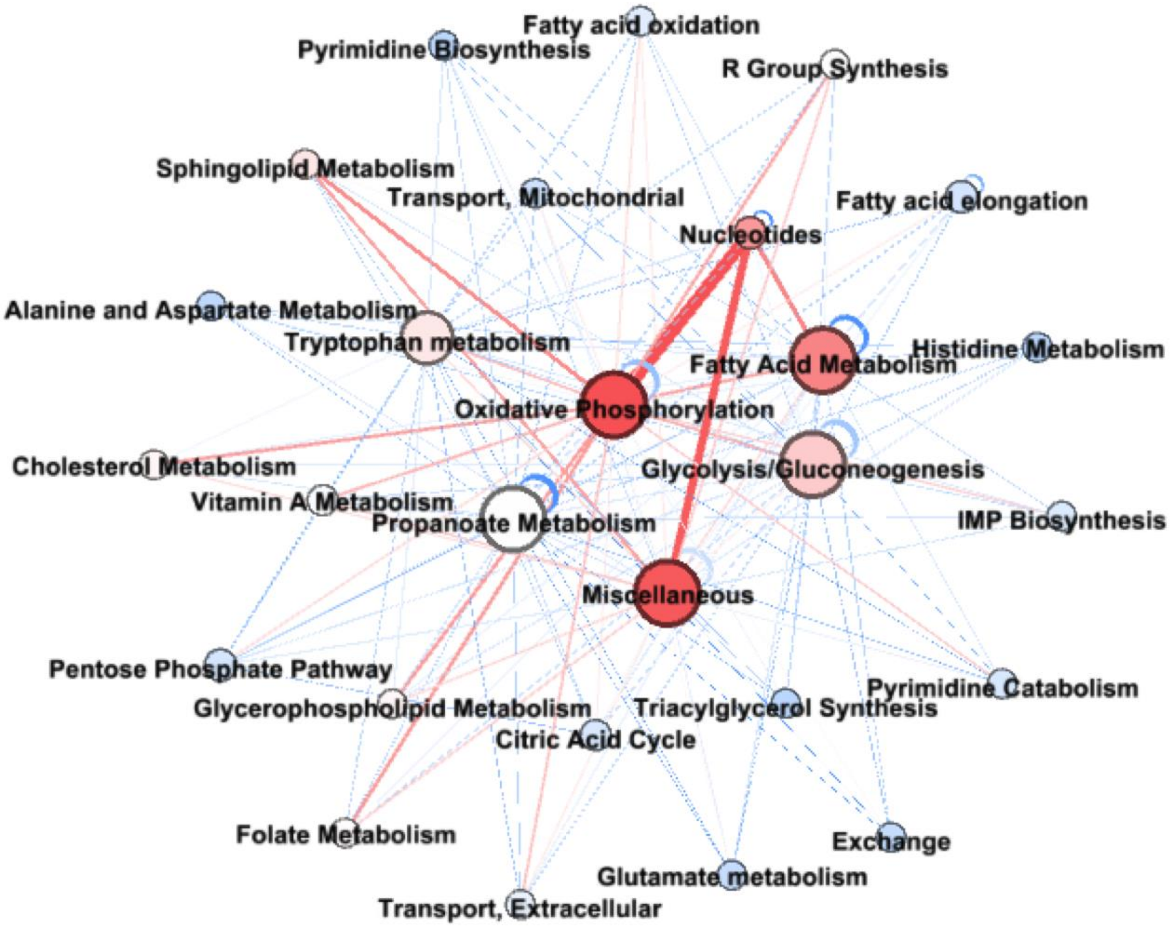

Supplementary Figure 3. Relationship between metabolic pathways, nodes represent pathways, the color and size of nodes represent the number of SLPs of genes with the corresponding pathway, the higher the number, the redder and larger of the node. Line represent the number of SLPs between the two pathways, the larger the number, the thicker the line. **(A)** Basal, **(B)** Lum B.
